# Dermatoscopic findings of syphilitic alopecia^☆^^☆☆^

**DOI:** 10.1016/j.abd.2020.01.007

**Published:** 2020-05-11

**Authors:** Izabella Cristina Cardozo Bomfim, Mayra Ianhez, Hélio Amante Miot

**Affiliations:** a Department of Dermatology, Hospital de Doenças Tropicais Dr. Anuar Auad, Goiânia, GO, Brazil; b Department of Tropical Medicine and Dermatology, Hospital das Clínicas, Universidade Federal de Goiás, Goiânia, GO, Brazil; c Sector of Psoriasis and Pediatric Dermatology, Hospital de Doenças Tropicais Dr. Anuar Auad, Goiânia, GO, Brazil; d Department of Dermatology and Radiotherapy, Faculdade de Medicina de Botucatu, Universidade Estadual Paulista, São Paulo, SP, Brazil

**Keywords:** Alopecia, Dermoscopy, Syphilis

## Abstract

Syphilis is an infectious disease that has afflicted mankind for centuries, but a recent increase in worldwide incidence has been evidenced. The authors describe a patient with typical lesions of secondary syphilis and moth-eaten alopecia, whose dermoscopic examination demonstrated empty hair follicles, vellus hair, follicular hyperkeratosis, peripheral black dots, dilated and tortuous vessels, reddish brown background, and hypopigmentation of the hair shafts. Furthermore, this case presented an erythematous background more evident than previously described cases.

## Introduction

Syphilis is an infectious disease that has afflicted mankind for centuries. Epidemiological data show that the incidence has increased over the last years throughout the world.^1^, ^2^ It is primarily a sexually transmitted disease. In addition, pregnant women can transmit the infection to their unborn child, characterizing congenital syphilis. The disease typically follows a progression through stages: primary syphilis (sore on or around the genitals), secondary syphilis (rash may appear as rough, red, or reddish brown spots both on the palms and soles, among other less common sites), and tertiary syphilis (affecting multiple organ systems, including the brain, nerves, heart, and blood vessels, among others).^1^, ^3^ The secondary stage may be accompanied by syphilitic alopecia (SA), whose prevalence may be underestimated due to its subtle presentation and difficult diagnosis. Dermoscopy is a useful tool to differentiate various hair diseases.^4^, ^5^, ^6^ The present report describes a patient with typical lesions of secondary stage and SA, whose dermoscopic diagnosis was compatible with other findings in the literature, but with the peculiarity of showing an erythematous background more evident than the cases previously described.

## Case report

**Figure 1:**
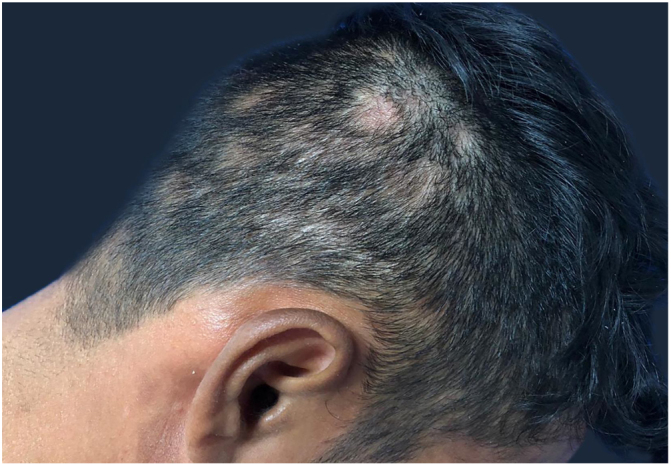
Areas of non-scarring alopecia on the scalp.

**Figure 2:**
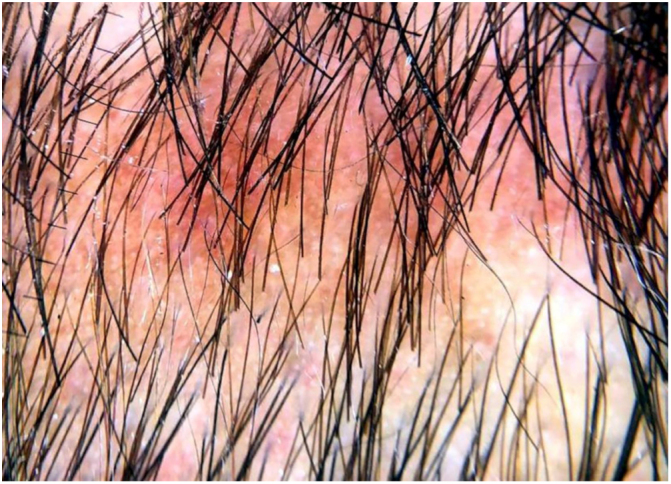
Dermoscopy of the areas of non-cicatricial syphilitic alopecia. * Dermatoscope: DermLite model DL3, ×10 magnification.

**Figure 3:**
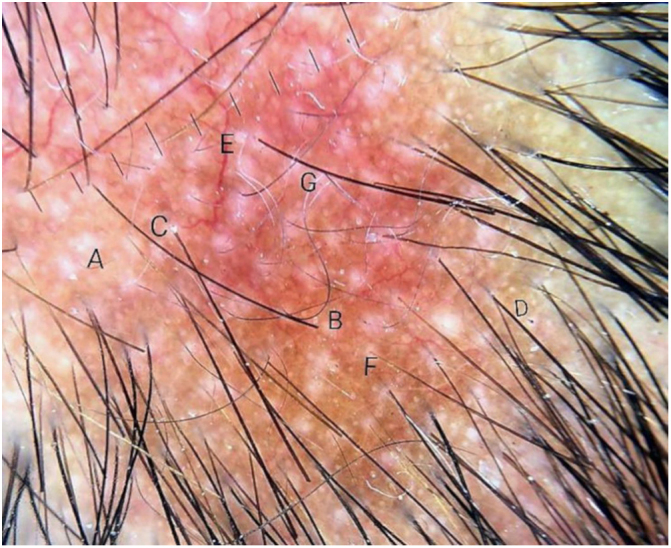
Dermoscopy of the areas of non-cicatricial syphilitic alopecia. A, Empty hair follicles; B, Vellus hair C, Perifollicular hyperkeratosis; D, Black point on the periphery; E, Dilated and tortuous vessels; F, Erythematous-brownish background; G, Hypopigmentation of the hair shafts.

A 29-year-old man, infected by HIV five years ago, had been using antiretroviral therapy with a history of poor adherence. His last CD4 demonstrated 674 cells and a viral load of 417 copies. He presented erythematous-brown, infiltrated plaques on the face, trunk, and arms, as well as areas of multiple non-healing alopecia, with poorly defined borders, present in the occipital region of the scalp (Fig. 1). A biopsy of the cutaneous lesion was performed. The anatomopathological examination described an intact epidermis, a dermis with superficial and deep perivascular neural inflammatory lymphohistiocytic infiltrate, presence of foci of inflammatory aggression to nervous filaments, and formation of epithelioid granulomas, without Langhans giant cells. He had been diagnosed with syphilis, with Venenearal Disease Research Laboratory test (VDRL) = 1/512. The dermoscopy (Figure 2, Figure 3) of the scalp lesions revealed empty hair follicles (3A), vellus hair (3B), follicular hyperkeratosis (3C), peripheral black dots (3D), dilated and tortuous vessels (3E), reddish-brown background (3F), and depigmented capillaries (3G). He was treated with benzathine penicillin, evolving with prompt regrowth in the areas of alopecia.

## Discussion

SA is an uncommon manifestation of syphilis infection. In 1940, McCarthy described two clinical types of SA, symptomatic and essential, the latter being divided into three patterns: moth-eaten or patchy alopecia, diffuse alopecia, and mixed-pattern alopecia. This classification continues to be used, practically unchanged. Although the clinical aspects of SA are well described, the trichoscopic findings have been demonstrated in scant publications in recent years, detailed in table 1.^4^, ^5^, ^6^, ^7^

**Table 1:** Dermoscopic findings of syphilitic alopecia described in the literature.

Author (year)	Number of patients	Dermoscopic findings
Tognetti et al. (2017)	1	Empty ostia and yellow dots are visible in the center of the alopecic patches over an erythematous background. Tapered bent hairs are present at the periphery of the alopecic patches. Vellus hairs are visible at the periphery. Scales appear to be thin and whitish; perifollicular hyperkeratosis is focally visible.
Doche et al. (2017)	3	Reduction of the number of hairs, yellow dots, broken and zigzag hair.
Piraccini et al. (2015)	4	Reduction in the number of terminal hairs and the presence of empty hair follicles, vellus hairs, red-brown background, and irregularly dilated capillaries with slight blood extravasation in four patients.
Ye et al. (2014)	1	Black dots, focal atrichia, hypopigmentation of hair shafts and yellow dots, and the scalp showed no obvious signs of inflammation or desquamation.
Review of all reported results	9	Yellow dots and black dots.
		Vellus hairs; curved, tapered, broken, or zigzag hairs.
		Hypopigmentation of the hair shaft.
		Empty hair follicles, reduction of terminal hairs.
		Dilated and tortuous vessels, slight extravasation of blood.
		Erythematous or erythematous-brownish background
		Perifollicular hyperkeratosis

Symptomatic SA is the most rare manifestation, in which there is an association of skin and scalp lesions, simultaneously. The essential SA is characterized by capillary loss, with no other visible syphilitic lesions. Moth-eaten alopecia is the most common, presented by multiple plaques of non-cicatricial alopecia, in the absence of local inflammation or scaling. They occur mainly in the parieto-occipital region, but may also arise in other areas such as beard, eyelashes, armpits, pubis, trunk, and legs. The diffuse SA is caused by capillary loss, like telogen effluvium. The mixed form is characterized by small irregular plaques that develop along with diffuse alopecia.^5^, ^8^, ^9^

SA has several differential diagnoses, such as alopecia areata (the main differential diagnosis of the patient in question), lupus, trichotillomania, tinea capitis, lichen planus pilaris, and telogen effluvium.^5^, ^8^ Clinical findings of cutaneous lesions associated with alopecia facilitate diagnosis, but cases with isolated alopecia may occur, making it difficult to diagnose SA.

Recent molecular studies have identified *Treponema pallidum* in the affected follicles, supporting the theory of a specific immune reaction to treponemal antigens. Immunohistochemistry can show the presence of spirochetes, generally in the peribulbar and perifollicular regions, indicating that this microorganism has a direct pathogenic role in alopecia.^10^

The patient in the reported case has presented the rarest form of alopecia, called symptomatic, due to the presence of skin and scalp lesions simultaneously. At dermoscopy, the typical signs described in the literature, such as vellus hairs, empty hair follicles, follicular hyperkeratosis, peripheral black spots, hypopigmented hairs, and dilated and tortuous vessels were visualized on an erythematous-brown background. However, the most striking feature was erythema more intense than the others reported, which may correspond to a symptomatic lesion of secondary syphilis. Trichoscopy can facilitate the diagnosis of SA in a patient with capillary loss of unknown origin.^5^, ^6^, ^8^ The clinical presentation, the serological screening for syphilis, and the histopathology of the scalp should be taken into account for the final diagnosis.

## Financial support

None declared.

## Authors’ contributions

Izabella Cristina Cardozo Bomfim: Approval of the final version of the manuscript; conception and planning of the study; drafting and editing of the manuscript; collection, analysis, and interpretation of the data; critical review of the literature; critical review of the manuscript.

Mayra Ianhez: Approval of the final version of the manuscript; conception and planning of the study; participation in the propaedeutic and/or therapeutic conduct of the cases studied; critical review of the manuscript.

Hélio Amante Miot: Approval of the final version of the manuscript; conception and planning of the study; critical review of the manuscript.

## Conflicts of interest

None declared.
